# Supplementary material for: Priming exposures to lipopolysaccharides do not affect the induction of Polycomb target genes upon re-exposure
Source: PLoS One. 2020 Apr 14;15(4):e0231498. doi: 10.1371/journal.pone.0231498 (PMC7156044; doi:10.1371/journal.pone.0231498)
Supplement: S1 File — (PDF) [file pone.0231498.s001.pdf]

Supporting Information

Priming exposures to lipopolysaccharides do not affect the induction of Polycomb target genes upon re-exposure

Marco Geigges [1], Geethika Arekatla [1], Renato Paro [1, 2 *]

1 Epigenomics Group, Department of Biosystems Science and Engineering, ETH Zurich, Basel, Switzerland
2 Faculty of Science, University of Basel, Basel, Switzerland

* Corresponding author
E-Mail: renato.paro@bsse.ethz.ch (RP)

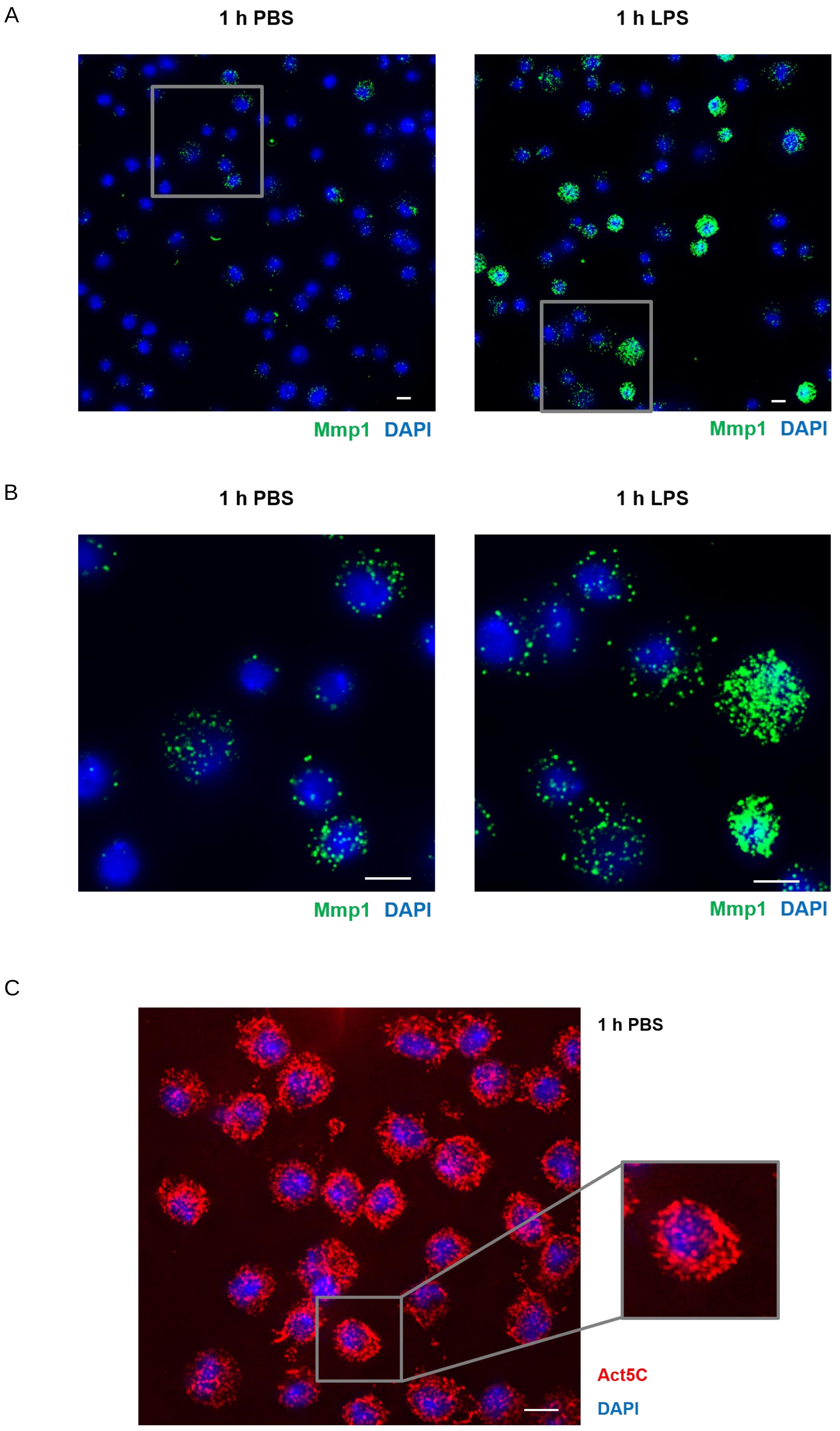
A
1 h PBS
1 h LPS
Mmp1 DAPI
Mmp1 DAPI
B
1 h PBS
1 h LPS
Mmp1 DAPI
Mmp1 DAPI
C
1 h PBS
Act5C
DAPI

Fig S1. Detection of individual endogenous Mmp1 and Act5C transcripts by fluorescent *in situ* hybridization in S2 cells.

(A, B) Cells were exposed to LPS for 1 h and subjected to *in situ* hybridization for Mmp1 transcripts. Control cells were treated with PBS only. Images in (B) represent a magnification of the sections highlighted in the images in (A).

(C) As a control for the *in situ* hybridization technique, cells were treated with PBS for 1 h and subjected to *in situ* hybridization for Act5C transcripts.

Scale bars: 5 µm.

Individual endogenous RNA transcripts were detected *in situ* in S2 cells using multiple probes each labeled by a single fluorophore (based on Raj et al. (2008)). For each target transcript, a set of up to 48 unique probes that collectively bind along an mRNA target molecule was designed. These fluorescently labelled probes produce one diffraction-limited spot per individual target mRNA. Thereby, individual RNA molecules can be visualized at the cellular level by fluorescence microscopy (Raj et al., 2006).

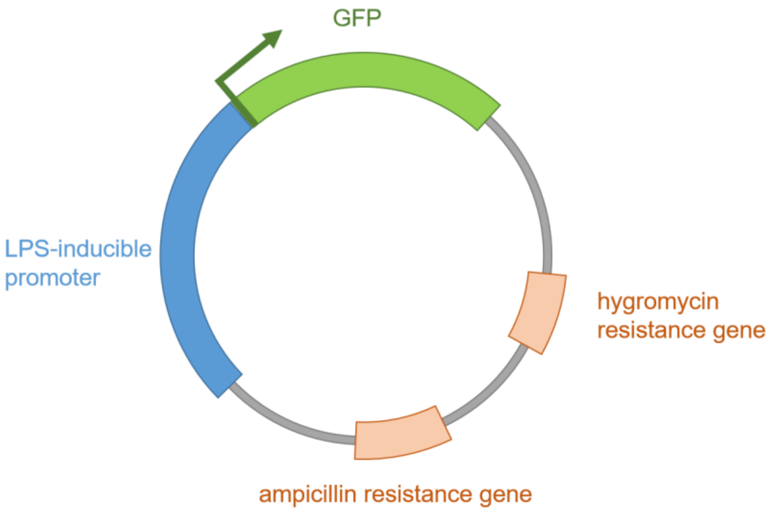

Fig S2. Schematic representation of the plasmid used to generate the reporter cell line. GFP expression is under the control of the LPS-inducible Mmp1 promoter. The vector contains an ampicillin resistance gene for amplification in bacteria and a hygromycin resistance gene for selection of stable S2 cells after transfection.

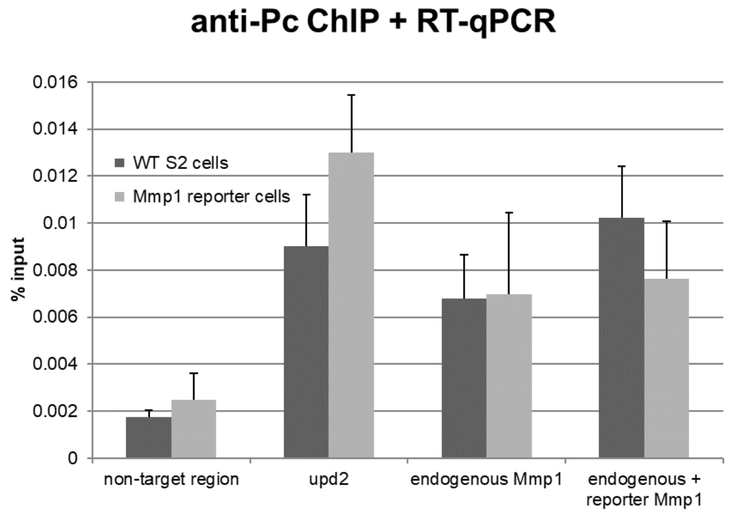

Fig S3. Polycomb (Pc) binding to candidate gene loci.

Anti-Pc chromatin immunoprecipitation (ChIP) in WT S2 and Mmp1 reporter cells followed by RT-qPCR. For Mmp1, two different primer pairs were used: a primer pair specific to the endogenous Mmp1 locus that does not amplify reporter sequences and primers that amplify a sequence bound by PcG proteins (as identified in the ChIP-Seq data) present in both the endogenous Mmp1 gene and the reporter construct. Results from three biological replicates are shown and error bars represent standard deviations.

Pc was bound to the endogenous Mmp1 locus to a similar extent in the Mmp1 reporter cell line as in WT S2 cells. Using a primer pair that amplifies a sequence in both the endogenous Mmp1 gene and the reporter construct, an input-normalized ChIP signal similar to the one for the primers specific to endogenous Mmp1 was detected in both cell lines. Comparably, the endogenous upd2 locus was similarly targeted by Pc in the two cell lines. This experiment verified that the Pc protein was bound to target loci equivalently in the Mmp1 reporter cell line and the WT S2 cells.

A

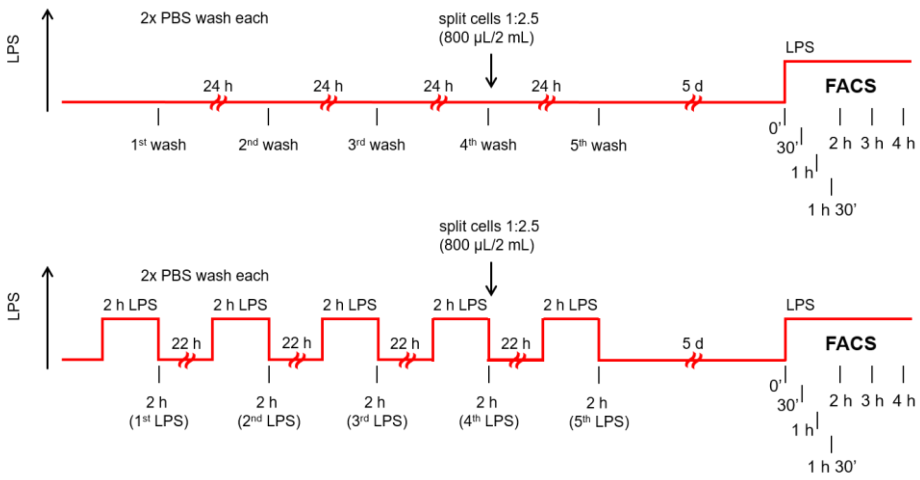
LPS
2x PBS wash each
split cells 1:2.5
(800 µL/2 mL)
24 h
5 d
LPS
FACS
1st wash
2nd wash
3rd wash
4th wash
5th wash
0'
30'
1 h
1 h 30'
2 h
3 h
4 h
2 h LPS
22 h
2 h
(1st LPS)
(2nd LPS)
(3rd LPS)
(4th LPS)
(5th LPS)

B

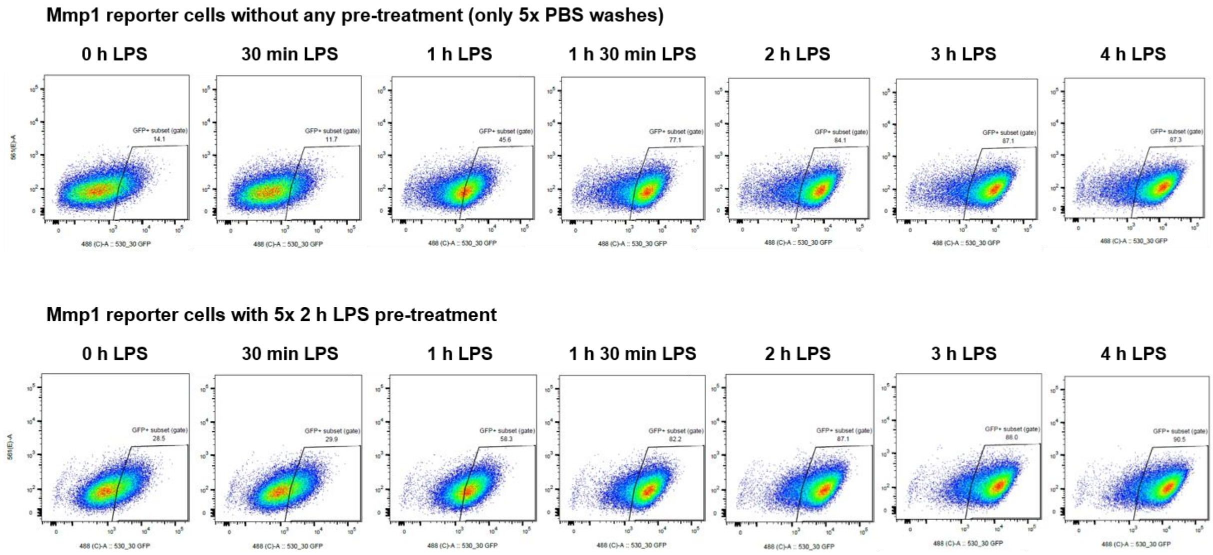
Mmp1 reporter cells without any pre-treatment (only 5x PBS washes)
0 h LPS
30 min LPS
1 h LPS
1 h 30 min LPS
2 h LPS
3 h LPS
4 h LPS
Mmp1 reporter cells with 5x 2 h LPS pre-treatment

C

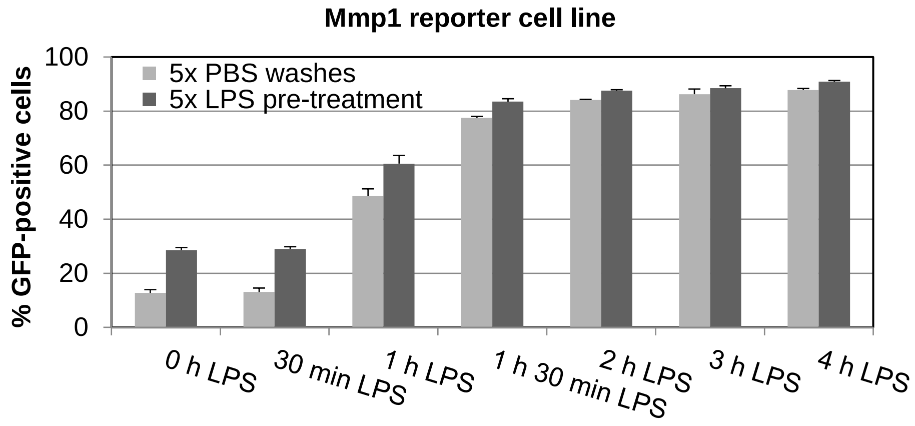
Mmp1 reporter cell line
% GFP-positive cells
5x PBS washes
5x LPS pre-treatment
0
20
40
60
80
100
0 h LPS
30 min LPS
1 h LPS
1 h 30 min LPS
2 h LPS
3 h LPS
4 h LPS

D

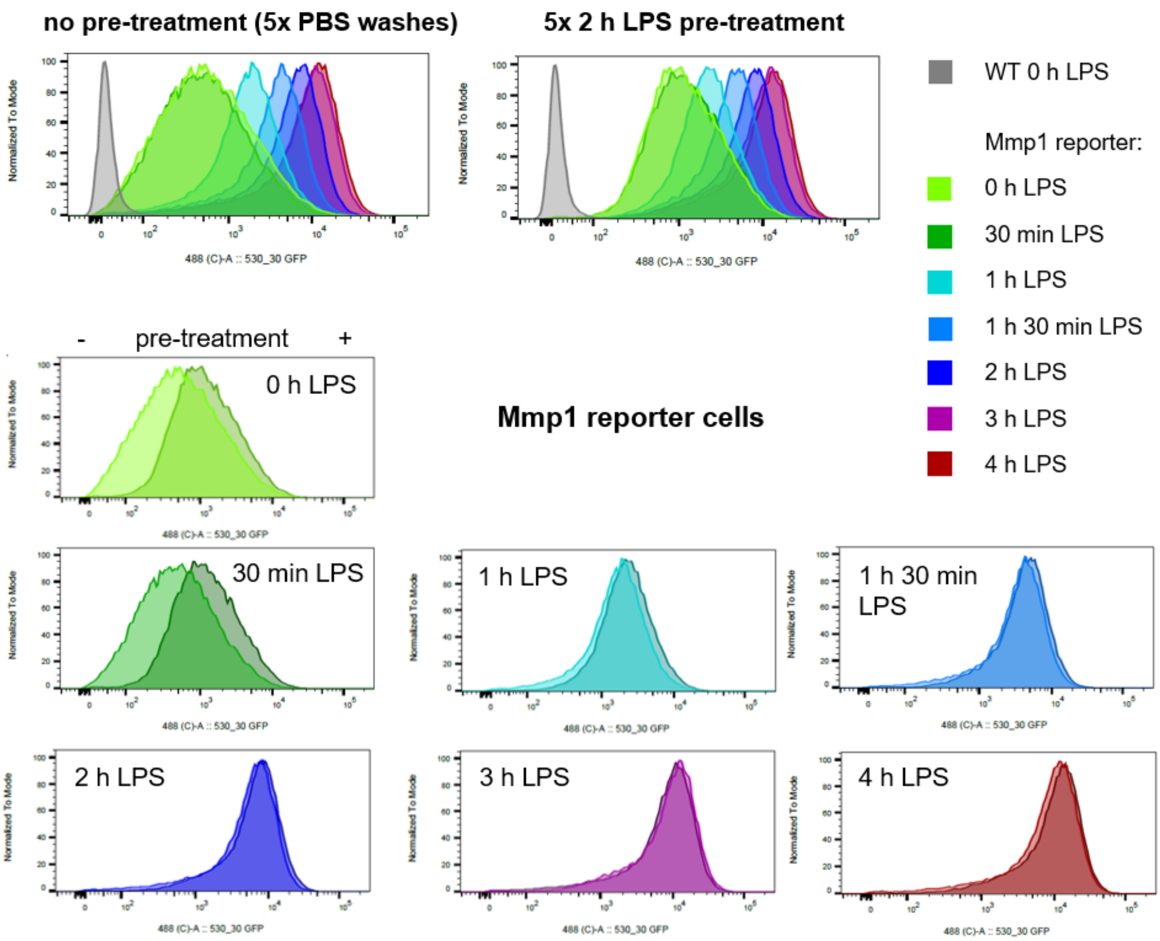
no pre-treatment (5x PBS washes)
5x 2 h LPS pre-treatment
WT 0 h LPS
Mmp1 reporter:
0 h LPS
30 min LPS
1 h LPS
1 h 30 min LPS
2 h LPS
3 h LPS
4 h LPS
- pre-treatment +
Mmp1 reporter cells
0 h LPS
30 min LPS
1 h LPS
1 h 30 min LPS
2 h LPS
3 h LPS
4 h LPS
Normalized To Mode
488 (C)-A :: 530_30 GFP

E

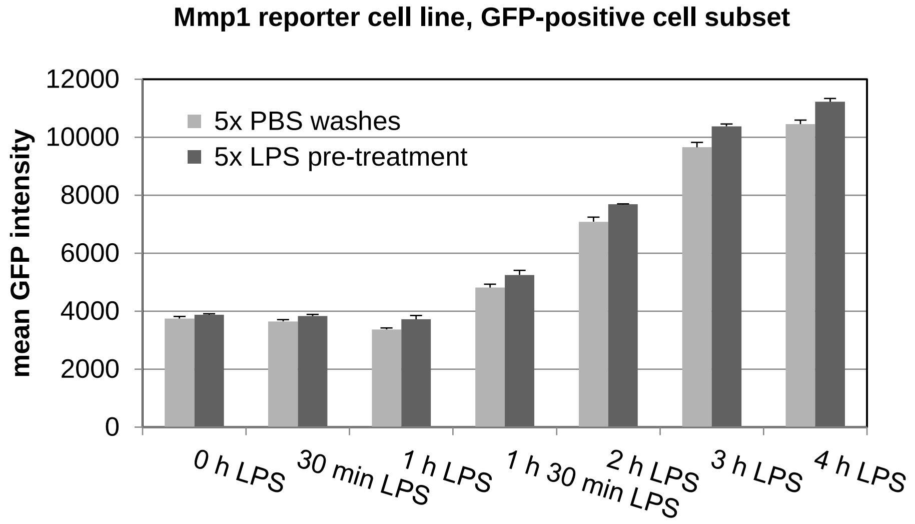
Mmp1 reporter cell line, GFP-positive cell subset
mean GFP intensity
12000
10000
8000
6000
4000
2000
0
5x PBS washes
5x LPS pre-treatment
0 h LPS
30 min LPS
1 h LPS
1 h 30 min LPS
2 h LPS
3 h LPS
4 h LPS

Fig S4. FACS analysis of Mmp1 reporter cells subjected to multiple LPS pre-treatment.

(A) Experimental outline of the multiple pre-treatment. Cells were stimulated for 2 h each on five consecutive days, further cultured, re-stimulated with LPS and subjected to FACS analysis.

(B) Dot plots of GFP fluorescence and (C) frequency of GFP-positive cells in reporter cells either without any pre-treatment or with multiple LPS pre-treatment are shown. Mean percentages from three biological replicates are given. Error bars represent standard deviations.

(D) The frequency distribution of the FACS data versus the GFP intensity is displayed in histograms for all time points of non-pre-treated and pre-treated cells and for each time point separately.

(E) The mean GFP intensity of the GFP-positive cell subset only is shown upon re-exposure of control and pre-treated cells. The geometric mean of the GFP intensity from three biological replicates was calculated and error bars represent standard deviations.

During the culturing period between the last pre-treatment and the start of the re-exposure, the GFP fluorescence, which had been induced by multiple pre-treatment, was not completely lost in all pre-treated cells. Regardless of this difference in the number of GFP-positive cells at the beginning of the re-exposure, no major differences were observed in GFP induction between reporter cells that have been pre-treated and the ones that have never been exposed before. For example, after 1 h 30 min of re-exposure, there were only 6% less fluorescent cells detected in the non-pre-treated culture than in the pre-treated one.

GFP intensity of reporter cells induced by re-stimulation, likewise, was overall not affected by the pre-treatment. At 0 min and 30 min of re-exposure, GFP intensity of reporter cells was stronger in pre-treated cells, reflecting residual fluorescence from the pre-treatment. However, already after 1 h of re-exposure, the GFP intensity distribution was the very same in both non-pre-treated and pre-treated cultures. When evaluating the mean GFP intensity in the GFP-positive cell subset only, there was no difference between pre-treated and control cells either.

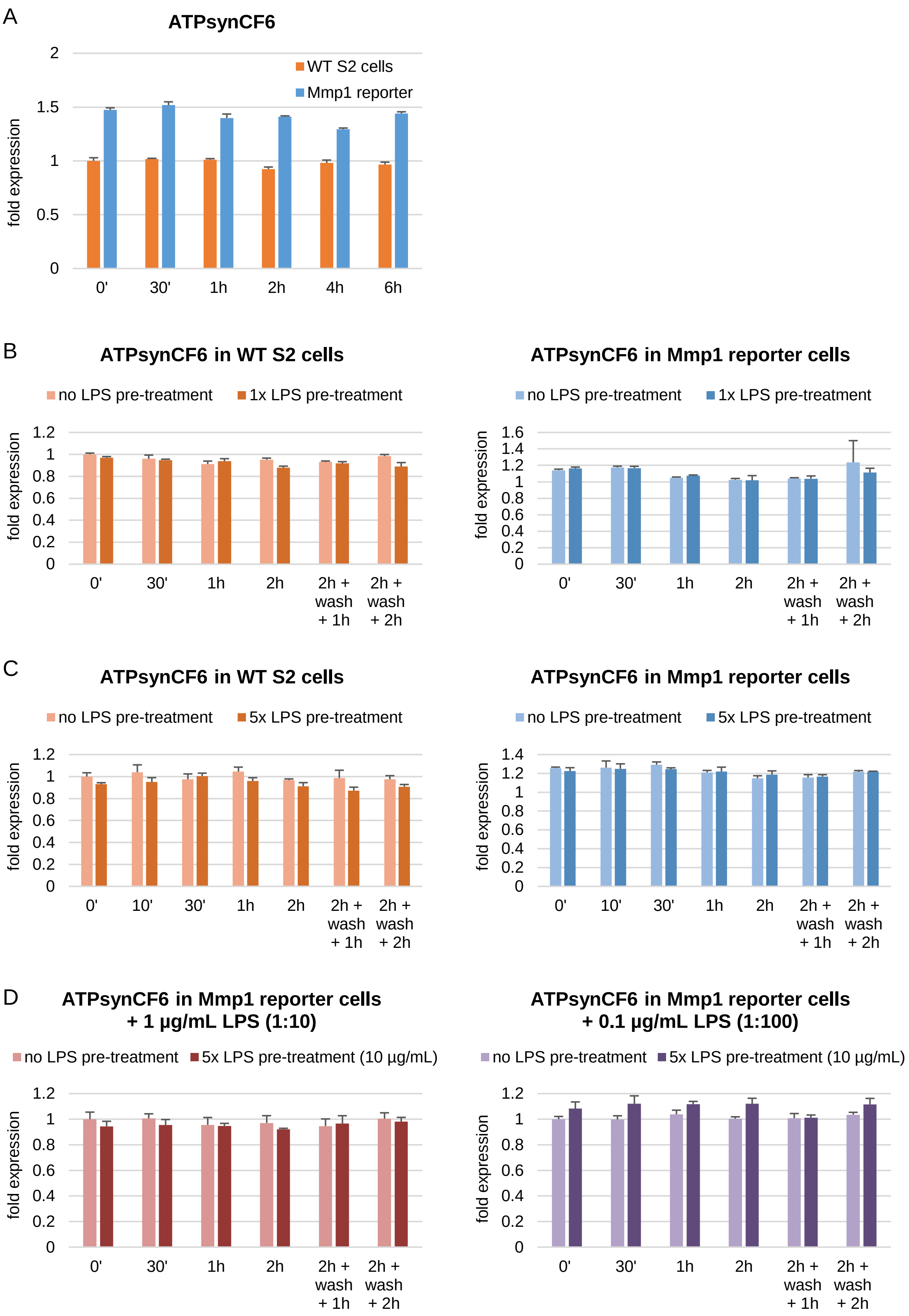

A
ATPsynCF6
WT S2 cells
Mmp1 reporter
fold expression
0' 30' 1h 2h 4h 6h
B
ATPsynCF6 in WT S2 cells
no LPS pre-treatment
1x LPS pre-treatment
ATPsynCF6 in Mmp1 reporter cells
no LPS pre-treatment
1x LPS pre-treatment
fold expression
0' 30' 1h 2h 2h + wash + 1h 2h + wash + 2h
C
ATPsynCF6 in WT S2 cells
no LPS pre-treatment
5x LPS pre-treatment
ATPsynCF6 in Mmp1 reporter cells
no LPS pre-treatment
5x LPS pre-treatment
fold expression
0' 10' 30' 1h 2h 2h + wash + 1h 2h + wash + 2h
D
ATPsynCF6 in Mmp1 reporter cells + 1 µg/mL LPS (1:10)
no LPS pre-treatment
5x LPS pre-treatment (10 µg/mL)
ATPsynCF6 in Mmp1 reporter cells + 0.1 µg/mL LPS (1:100)
no LPS pre-treatment
5x LPS pre-treatment (10 µg/mL)
fold expression
0' 30' 1h 2h 2h + wash + 1h 2h + wash + 2h

Fig S5. Expression of ATPsynCF6 in WT and Mmp1 reporter cells subjected to different LPS treatments.

In all RT-qPCR experiments, ATPsynCF6 transcript levels were evaluated in respect to RpL32 expression. Expression levels were normalized to the 0 min time point values in WT S2 cells that were not pre-treated by LPS. Robustly constant expression of ATPsynCF6 transcripts as a second reference gene product verified the usage of RpL32 as a reference gene in all experiments shown. ATPsynCF6 expression in (A) is related to experiments depicted in Figure 2, (B) related to Figure 3, (C) related to Figure 4 and (D) related to Figure 5.

Table S1. Primer pairs used in this study for RT-qPCR experiments.

Gene Symbol	Gene Name	Annotation Symbol	Primer Name	Primer Sequence
ATPsyn CF6	ATP synthase, coupling factor 6	CG4412	MGP13a<br>MGP13b	F: 5'-AGA AGA GCG CCG GTG GCA A-3'<br>R: 5'-AAC ATC GGG GAA CTG GAA TTC G-3'
CecA1	Cecropin A1	CG1365	MGP023<br>MGP024	F: 5'-GAA CTT CTA CAA CAT CTT CGT-3'<br>R: 5'-TCC CAG TCC CTG GAT T-3'
GFP	Green Fluorescent Protein	-	MGP160<br>MGP161	F: 5'-ATC ATG GCC GAC AAG CAG AA-3'<br>R: 5'-TCT CGT TGG GGT CTT TGC TC-3'
Mmp1	Matrix metallo-proteinase 1	CG4859	MGP001<br>MGP002	F: 5'-TGA TGG AAA ACA GGG TGC CT-3'<br>R: 5'-CCC ACT GCA AAA CCA CCA AC-3'
PGRP-LF	Peptidoglycan recognition protein LF	CG4437	MGP21a<br>MGP21b	F: 5'-CAG GGC ATA AAA CTT GGT CA-3'<br>R: 5'-GAA AAA TGG CAC AGG AAT GG-3'
puc	puckered	CG7850	MGP033<br>MGP034	F: 5'-TGG CTC TGT TCA AGC G-3'<br>R: 5'-CCT TAT CTC AGT CCC TCG-3'
RpL32	Ribosomal protein L32	CG7939	MGP035<br>MGP036	F: 5'-GAC GCT TCA AGG GAC AGT ATC TG-3'<br>R: 5'-AAA CGC GGT TCT GCA TGA G-3'
upd2	unpaired 2	CG5988	MGP007<br>MGP008	F: 5'-CAC GAC GCT CAG GAT CAT GA-3'<br>R: 5'-CCT TAA ACG CCA GCC AAC AG-3'

Table S2. Induction levels of selected LPS-inducible PcG target genes (see Fig 2).

Gene Symbol	Gene Name	Annotation Symbol	Fold Expression in WT S2 Cells		
			30 min LPS	1 h LPS	2 h LPS
Mmp1	Matrix metalloproteinase 1	CG4859	4.7	9.0	5.0
PGRP-LF	Peptidoglycan recognition protein LF	CG4437	2.5	4.8	5.3
puc	puckered	CG7850	1.6	3.3	1.1
upd2	unpaired 2	CG5988	3.3	3.7	1.1

Supplementary Materials and Methods

Generation of the GFP reporter plasmid

For the generation of the reporter cell line, a plasmid containing a GFP reporter whose expression is under the control of the Mmp1 promoter was constructed. The Mmp1 promoter region was amplified from genomic DNA of S2 cells with primers MGP051 and MGP052 (Table S3) and Q5 High-Fidelity DNA Polymerase (New England Biolabs, USA) according to manufacturer's instructions without using the enhancer. PCR products were purified with QIAquick PCR Purification Kit (Qiagen, Germany) as suggested by the manufacturer, integrated into the TOPO Zero Blunt vector using Zero Blunt TOPO PCR cloning kit (Life Technologies, Thermo Fisher Scientific, USA) according to manufacturer's suggestions and transformed into competent TOP10 *E. coli* cells. Plasmids with promoter sequences were isolated with GenElute Plasmid Miniprep Kit (Sigma-Aldrich, Switzerland) according to manufacturer's instructions and verified by sequencing (Microsynth, Switzerland).

As a fluorescent reporter gene, GFPmut3, a variant of GFP characterized by a faster maturation rate than the enhanced green fluorescent protein (EGFP), was used. The GFPmut3 sequence for the reporter construct was generated by exchanging the amino acids L64F, T65G and S72A of EGFP present in a pGEM T-Easy vector (Promega, USA) by site-directed mutagenesis with phosphorylated primers MGP037 and MGP038 (Table S3). GFPmut3 PCR products were circularized by T4 DNA Ligase and EGFP templates were digested by DpnI. Cloning products were transformed into competent TOP10 *E. coli* cells, amplified, isolated and verified by sequencing.

As a vector backbone for the reporter construct, sequences from the pMT-FHW plasmid (*Drosophila* Gateway Vector collection) were used. By site-directed mutagenesis of the pMT-FHW plasmid with phosphorylated primers MGP072 and MGP073 (Table S3), its Gateway Cassette was deleted, HygroR and AmpR resistance genes were retained and unique restriction enzyme sites ApaI, EcoRV, SphI and StuI were inserted.

The Mmp1 promoter fragment and the GFPmut3 sequence were inserted into this vector backbone by Gibson Assembly. For this purpose, the Mmp1 promoter sequence was amplified from the generated TOPO Zero Blunt vector using primers MGP078 and MGP079 (Table S3). The GFPmut3 sequence was obtained from the created pGEM T-Easy vector with primers MGP074 and MGP075 (Table S3) by PCR. These PCR mixes were separated on 0.8% (w/v) agarose gels and desired fragments were extracted and purified with the QIAquick Gel Extraction Kit (Qiagen, Germany) following the manufacturer's instructions. The vector backbone plasmid with the HygroR resistance gene was linearized with EcoRV and SphI and GFPmut3 and promoter fragments were integrated using Gibson Assembly Master Mix (New

England Biolabs, USA) as suggested by the manufacturer. The sequence of the reporter plasmid was verified by sequencing (Microsynth, Switzerland).

Table S3. Primer pairs used in this study to generate the reporter plasmid.

Primer Name	Purpose	Primer Sequence (5'-3')
MGP037	site-directed mutagenesis of EGFP sequence to generate GFPmut3 sequence	CGT AGC CGA AGG TGG TCA CGA GGG TGG G
MGP038		GCG TGC AGT GCT TCG CCC GC
MGP051	amplification of Mmp1 promoter region from genomic DNA	TAT CTA ACC ACC AAG ATC TAA T
MGP052		CTA CGA CTA TGA ACA CTG AAC T
MGP072	site-directed mutagenesis of pMT-FHW plasmid	AAT GCA TGC TAA AGG CCT AAT TGA TCA GTC GAC TCT AGA GGA TCC
MGP073		ATT GAT ATC TTA GGG CCC ATT GGC GCC TGA TGC GGT ATT T
MGP074	amplification of GFPmut3 sequence for Gibson Assembly	GTG AGC AAG GGC GAG GA
MGP075		GAT CCT CTA GAG TCG ACT GAT CAA TTA GGC CTT TAG CAT GCA TTA ATT CAT TAA TCG GAA TTG CGG CCG
MGP078	amplification of Mmp1 promoter sequence for Gibson Assembly	AGG AGA AAA TAC CGC ATC AGG CGC CAA TGG GCC CTA AGA TAT CAA TAC CAC CAA GAT CTA ATC GCC ATC G
MGP079		TGG GCA CCA CCC CGG TGA ACA GCT CCT CGC CCT TGC TCA CCA TTT TGT TGC TGT TGT TGC TGT TG

RNA FISH probes

RNA FISH probes were designed for *Drosophila* Act5C and Mmp1 transcripts using the Stellaris RNA FISH Probe Designer (Biosearch Technologies, USA) available online at www.biosearchtech.com/stellarisdesigner (version 4.1). Target sequences for probe design of Mmp1 were cleared from sequences identical to Mmp2. For each gene, at least 44 fluorescently labelled probes of 20 nt each were ordered from Stellaris (Biosearch Technologies, USA) (Table S4, Table S5). FISH probes for Mmp1 were marked with Quasar 570 Dye and the Act5C probes with Quasar 670 Dye. 5 nmol probes were dissolved in 200 µL TE (pH 8.0), directly aliquoted into RNAse-free tubes and stored at -20°C.

Table S4. RNA FISH probes for Act5C.

Probe #	Probe Sequence (5'-> 3')	Probe Position*	GC Content [%]
1	agcacaagaactcaaacggt	13	45.0
2	tttgtgtcgggaggagtatc	38	50.0
3	tagacgactgctggctgatg	65	55.0
4	ctttcggtttggtgtctctg	88	50.0
5	ttcttcgtcacacattttgt	157	35.0
6	gttgtcgacaaccagagcag	181	55.0
7	acaatcgatgggaagacggc	249	55.0
8	gtacttcagggtgaggatac	352	50.0
9	tcacgataccgtgctcaatg	374	50.0
10	cagatcttctccatatcgtc	402	45.0
11	acacgcagctcattgtagaa	432	45.0
12	tgatctgggtcatcttctca	512	45.0
13	ggtgtgttgaaggtctcgaa	534	50.0
14	ctggatggccacatacatgg	556	55.0
15	cagaacgataccggtggtac	604	55.0
16	aagggcataaccctcgtaga	658	50.0
17	aaatcgcgaccagccagatc	699	55.0
18	aggatcttcatcaggtagtc	723	45.0
19	gtggtgaaagagtaaccgcg	750	55.0
20	atgtcacggacgatttcacg	780	50.0
21	caacatagcacagcttctcc	803	50.0
22	atctcctgctcaaagtcgag	825	50.0
23	taggacttctccaacgagga	864	50.0
24	caagaacgagggctggaaca	946	55.0
25	atgatggagttgtaggtggt	993	45.0
26	acggatatccacatcacact	1015	45.0
27	acggtgttggcatacagatc	1038	50.0
28	gtgatctccttctgcatacg	1098	50.0
29	tcttgatcttcatggtcgac	1130	45.0
30	cgatccagacagagtacttg	1169	50.0
31	acagcgaagccaggatggag	1193	60.0
32	agatccacatctgctggaag	1217	50.0
33	acttgcggtgcacaatggag	1265	55.0
34	gacaagcgatccttcttaga	1288	45.0
35	agaatcaagaccatcccgat	1325	45.0
36	cactcgcacttgcactttcg	1382	55.0
37	ttgtgctgcactccaaactt	1406	45.0
38	tccgctcttttcatcttgta	1448	40.0
39	acacaacatgcgcccaaaac	1500	50.0
40	tcatatcatatctcatgtgg	1551	35.0
41	tcattttttgtagttcttgt	1619	25.0
42	ggcagaatatgcattttcgc	1645	45.0
43	atcttgatgtccttgtcatg	1732	40.0
44	gcgctttgggaaatatcttc	1767	45.0
45	tgcaatgtgtgtgtgttctt	1790	40.0
46	acgctagtgtgttgtttgtg	1813	45.0
47	ggttgctgacgaattgtaca	1835	45.0
48	attttcctttttgttttcat	1908	20.0

*Position of first nucleotide of probe corresponding to Act5C-RD mRNA sequence

Table S5. RNA FISH probes for Mmp1.

Probe #	Probe Sequence (5'-> 3')	Probe Position*	GC Content [%]
1	gctggcactttgaactttat	5	40.0
2	cgtctatttgtcatgtggtt	34	40.0
3	aactttggcagtttgtcatt	126	35.0
4	gtgcctacgactatgaacac	148	50.0
5	cgctgccatgatggaaaaca	170	50.0
6	tggaaacgggtgccgattga	192	55.0
7	caggtagatctctgcctgtg	218	55.0
8	cgggtagatagccgaactgg	240	60.0
9	ttggaactcctcgatggcac	314	55.0
10	tgatgttcagtcccgcgaag	336	55.0
11	gacatcagcttcattgtctc	373	45.0
12	taggtgaggttcttcacacg	475	50.0
13	cttggggtacttggagatct	497	50.0
14	tcgacatcgactcgcttcag	520	55.0
15	ctcggaccacacggcaaatg	557	60.0
16	gtcttcctggtgaaggtcag	586	55.0
17	tccacgaacttgatctcgat	622	45.0
18	cttgaccgtcaaaggcatcg	660	55.0
19	agacagggaagaaggcatgc	693	55.0
20	acagttccgcgtcgtcaaag	715	55.0
21	gaacagattggtgcctcgag	750	55.0
22	aaggagtgaccgaactcgtg	782	55.0
23	ctctgatctgagtgggagag	806	55.0
24	tcaaagccgcgatagaaggg	842	55.0
25	ctcgtccaacttgaagacgg	864	55.0
26	catacagtgactggatggcc	895	55.0
27	ctcagctggtttgttttgcg	917	50.0
28	cacctttggaggagagtatg	972	50.0
29	ttgcagatggagtcgtctag	995	50.0
30	agttgaagagtgtgtctacc	1024	45.0
31	tcagcttgtagtacttgtcg	1075	45.0
32	tggagatgagctgtgggtag	1117	55.0
33	tgaacgccgcatcgatgttg	1159	55.0
34	gtagcgccagtactgagtac	1196	55.0
35	ctgatttccttagggtagac	1237	45.0
36	cgtccagatggtcgggaatg	1272	60.0
37	gcccttgaagaagtatatct	1319	40.0
38	gatcgaagcgccagaacttg	1341	55.0
39	tggagatgggcttgggatag	1389	55.0
40	gtccaggttgttgggaacac	1418	55.0
41	tagccattggtgtacttcag	1444	45.0
42	tgtcgcccttgaagaagtac	1467	50.0
43	gcgtcatggaatctgtagta	1489	45.0
44	tggccgagtcaacagcaaat	1512	50.0

*Position of first nucleotide of probe corresponding to common Mmp1 mRNA sequence of all isoforms depleted by sequence stretches identical to Mmp2

Detection of individual endogenous RNA transcripts by *in situ* hybridization in S2 cells

For FISH experiments, 1 million S2 cells in a volume of 1 mL per well were seeded into 12-well plates with poly-L-lysine-coated cover glasses. One day after seeding, cells were treated with PBS or LPS. Following two washes with 1x PBS, cells were incubated with 1 ml fixation solution (4% (w/v) paraformaldehyde in 1x PBS) per well for 10 min at room temperature. They were washed two more times in 1x PBS and permeabilized in 70% ethanol at 4°C overnight. Afterwards, cells were washed twice with 1x PBS and twice with wash buffer (2x SSC, 10% (v/v) formamide in nuclease-free water) for 5 min each while gently shaking.

For hybridization, a cover glass with cells was placed upside-down on top of a drop of 45 µl hybridization solution. Hybridization solution was composed of 1 µl of each FISH probe in 200 µl hybridization buffer (10% (w/v) dextran sulfate sodium salt from *Leuconostoc* spp. (Sigma-Aldrich, Switzerland), 2x SSC, 10% (v/v) formamide in nuclease-free water). Hybridization reactions were incubated at 37°C in the dark for 4 h. Afterwards, they were washed twice for 30 min each with wash buffer warmed up to 37°C. These two washes were carried out in the dark while gently shaking. Wash buffer was aspirated and 1 mL DAPI nuclear stain solution (500 ng/mL in 1x PBS) was added per well. DAPI solution was immediately removed and cells were washed twice in 1x PBS. A drop of ProLong Gold Antifade Mountant (Thermo Fisher Scientific, USA) was put on a glass slide and a cover glass with cells was placed upside-down on top of it. Samples were kept overnight. They were sealed and imaged on a Nikon Ti-E inverted wide field microscope (Nikon Instruments Europe, Netherlands) the next day.

Chromatin immunoprecipitation

For ChIP experiments, S2 WT and Mmp1 reporter cells were cultured in 145 mm plates (Cellstar Cell Culture Dishes, Greiner Bio-One, Austria). Cells were washed once in PBS and incubated with 10 ml fixation solution (50 mM KOH, 1 mM EDTA, 0.5 mM EGTA, 100 mM NaCl, 100 mM $NaHCO_3$, 1% methanol-free formaldehyde (Thermo Fisher Scientific, USA)) at room temperature for 6 min while gently shaking. 125 mM glycine was added to the plates afterwards and cells were incubated at room temperature for an additional 5 min.

Cells were then scraped off the plates and pelleted at 4°C. Pellets were resuspended in 10 ml nuclear isolation buffer (256 mM sucrose, 8 mM Tris pH 8 at 4°C, 1 mM EDTA, 0.5 mM EGTA, 0.8% Triton X100, 1 µg/mL Leupeptin, 1 µg/mL Pepstatin A, 1 µg/mL Aprotinin). Cell suspensions were rotated at 4°C for 10 min followed by centrifugation at 4°C. Pellets were then resuspended in 1 ml sonication buffer (10 mM Tris pH 8 at 4°C, 1 mM EDTA, 0.1% SDS) supplemented with cOmplete protease inhibitors (Roche Life Science, Switzerland). After 5 min on ice, samples were transferred into milliTubes (Covaris, USA) and sonicated in an S220 focused-ultrasonicator (Covaris, USA) for 13 minutes at 5% duty cycle, 200 cycles per burst and 140 W maximal incidence power.

After sonication, 250 µL 5x dilution buffer (10 mM Tris pH 8 at 4°C, 1 mM EDTA, 750 mM NaCl, 5% Triton X-100, 0.1% SDS, 0.5% sodium deoxycholate) supplemented with cOmplete protease inhibitors were added. Samples were kept on ice for 5 min and subsequently centrifuged at 4°C for 10 min. Supernatants were taken, aliquoted, flash-frozen using liquid nitrogen and stored at -80°C until further processed.

For immunoprecipitation, chromatin aliquots were thawed on ice and pre-cleared with Dynabeads Protein A (Thermo Fisher Scientific, USA) that had been washed three times in wash buffer 1 (10 mM Tris pH 8 at 4ºC, 1 mM EDTA, 150 mM NaCl, 1% Triton X-100, 0.1% SDS, 0.1% sodium deoxycholate). After rotating at 4°C for 20 min, beads were removed and 5 µL anti-Pc antibody (Zink and Paro, 1989) was added per 120 µL chromatin sample. Samples were rotated at 10 rpm at 4°C for 16 hours.

Beads were subsequently washed five times in 400 µL wash buffer 1, once in 400 µL wash buffer 2 (10 mM Tris pH 8 at 4°C, 1 mM EDTA, 500 mM NaCl, 1% Triton X-100, 0.1% SDS, 0.1% sodium deoxycholate) and finally once in 400 µL TE, for 8 minutes each at 4°C.

For elution, beads were resuspended in 100 µL elution buffer (1% SDS, 100 mM $NaHCO_3$) and incubated at 65°C for 15 min while shaking at 800 rpm. This elution step was repeated two more times. 12 µL 5 M NaCl were added to the combined elution samples. For subsequent de-crosslinking, samples were incubated at 65°C overnight. RNA was removed by incubation with 8 µL RNase A (Roche Life Science, Switzerland) at 37°C for 90 min. 3 µL 20 mg/mL Proteinase K (Roche Life Science, Switzerland) were added and samples were incubated at 55°C for 60 min.

DNA was purified using the QIAquick PCR Purification Kit (Qiagen, Germany) as recommended by the manufacturer. ChIP samples were analyzed by RT-qPCR (see Table S6 for primers used) and results for each target locus were normalized to the respective input samples.

Table S6. Primer pairs used in this study for ChIP + RT-qPCR experiments.

Primer Name	Target	Primer Sequence (5'-3')
MGP165a	Pc peak around Mmp1 TSS, specific primer pair for endogenous Mmp1 locus	TAT CGG GCT TAT CTG CGA CC
MGP166a		CGT AGC CAA ATT CGC CCA AG
MGP167a	Mmp1 promoter sequence present both at the endogenous locus and the reporter gene	TGG AGA GGA GGC GTA TGA GT
MGP168a		AGG GAA GCG GGG AGA GTA AT
MGP169a	Pc peak upstream of upd2 TSS	TCG ACT CGG CTC GAC TCA T
MGP170a		CTC AAG GCC TCG AAC GGG
MGP171a	non-target region, downstream of bx PRE region	CGA ATG CGT CCC TCT CAT CA
MGP172a		TGG AAG GGC GTT GAC ATC TC

Supplementary References

Raj, A., Peskin, C.S., Tranchina, D., Vargas, D.Y., and Tyagi, S. (2006). Stochastic mRNA synthesis in mammalian cells. PLoS biology 4, e309.

Raj, A., van den Bogaard, P., Rifkin, S.A., van Oudenaarden, A., and Tyagi, S. (2008). Imaging individual mRNA molecules using multiple singly labeled probes. Nature methods 5, 877-879.

Zink, B., and Paro, R. (1989). In vivo binding pattern of a trans-regulator of homoeotic genes in Drosophila melanogaster. Nature 337, 468-471.
