# Supplementary material for: Priming exposures to lipopolysaccharides do not affect the induction of Polycomb target genes upon re-exposure
Source: PLoS One. 2020 Apr 14;15(4):e0231498. doi: 10.1371/journal.pone.0231498 (PMC7156044; doi:10.1371/journal.pone.0231498)
Supplement: Attachment — Submitted filename: repsonse_to_reviewers.docx [file pone.0231498.s002.docx]

**Revision of Manuscript PONE-D-19-32589 – Response to Reviewers**

We would like to gratefully thank both reviewers and the editor for their interest in our manuscript and their valuable contributions towards its improvement.

The manuscript underwent revision to comply with reviewers’ comments. Below, is our point-by-point response to reviewers’ comments.

**In response to Reviewer 1:**

*Reviewer #1: The manuscript by Geigges et al. follows a clear hypothesis of the epigenetic Polycomb (Pc) gene regulatory system mediating an adaptive innate immune response in Drosophila S2 cells. To test this idea, several target genes of LPS exposure were identified on the basis of previously published studies. Since the authors found the fluorescence detection of responses to LPS stimulus by FISH analysis not sensitive enough, a reporter cell line carrying a GFP downstream of one of the putative Pc regulated genes of innate immune response was generated. In multiple schemes of treatment and exposure to LPS, the authors did not detect an adaptive response of Pc target genes to LPS treatment in S2 cells.*

*While the overall results of the study are negative, the experimental schemes are generally carefully thought out and executed. The work provides a clear conclusion that can be of value to the scientific community.*

*Criticism:*

*The title of the manuscript reflects the experiments but not the results of the work.*

**Response:** We agree with reviewer 1 and have changed the title of the manuscript to better reflect the results of our work: “Priming exposures to lipopolysaccharides do not affect the induction of Polycomb target genes upon re-exposure”.

*I found the abstract confusing and difficult to read. It took me some time to comprehend what the work is about.*

**Response:** We thoroughly revised the abstract to introduce our work more clearly and to summarize our findings more comprehensibly.

*The authors use a single reference for RT-PCR quantification and normalization. A second control is mentioned but the results are not standardized to more than one reference. As references can be deceiving, most studies make use of multiple references for RT-PCR analysis and this has become a standard for this kind of experiment.*

**Response:** We agree that references in RT-qPCR analyses can be deceiving. Therefore, we did not use RpL32 as a single reference in our study, but also analyzed the expression of ATPsynCF6 as an additional endogenous reference gene in each and every RT-qPCR experiment performed in this project. The expression of our genes of interest was normalized to RpL32 levels and the constant expression of RpL32 across all samples was verified by quantification relative to ATPsynCF6. For details, see Fig S5 (named Fig S4 in the initial submission). The constant expression of both RpL32 and ATPsynCF6 justifies our normalization approach and excludes any potential false conclusions from deceiving references.

*The authors identify Mmp1 as a suitable reporter for their treatment scheme on the basis of its induction level. A) The induction levels should be specified in table 1 for a reader to have all this information.*

**Response:** We acknowledge that the induction levels are not specified in the original manuscript. From Boutros, M. *et al.* (2002) and from our own analyses, induction levels are not available for all genes listed in Table 1. Therefore, we have added the induction levels of LPS-inducible PcG target genes as a new Table S2.

*B) While this rationale seems fair, why were other or better additional targets not considered for constructing reporters? A single reporter might introduce a bias into the study.*

**Response:** We have considered additional LPS-inducible PcG target genes for the generation of reporter cell lines. In the end, however, we decided to focus on Mmp1 as it was found to be the top inducible LPS target gene of the immediate early group in the transcriptomic analysis. For this reason, a potential memory effect upon repeated LPS exposure would have been largest for Mmp1. A large absolute difference in induction levels between pre-treated cells and cells that have never been exposed to LPS before would have been ideal to further study such a transcriptional memory, if it existed. Additionally, the promoter region of Mmp1 to be used for reporter gene regulation has been shown before to be functional (see also our response to the next point) which was not the case for other LPS-inducible PcG target genes. Therefore, it would have been very difficult to decide on a promoter region of other genes for the construction of additional reporters and we would have needed to first characterize these promoters in detail.

*C) Only the promoter region of Mmp1 was included in the reporter construct. What is the rationale for considering this region a sufficient element for mediating the LPS effect? The authors mention in the discussion that distal gene regulatory elements could be involved in a putative adaptive immune response.*

**Response:** It has been shown before that the 4.78 kb Mmp1 promoter sequence used to generate the reporter construct in this study is sufficient for the transcriptional regulation of Mmp1 expression (Uhlirova, M. & Bohmann, D., The EMBO Journal, 2006). This region includes the three AP-1 binding sites described to regulate Mmp1 expression. Furthermore, this promoter sequence has been used in the past to generate several independent transgenic reporter flies in which it regulates the expression of a *lacZ* reporter gene. Reporter expression in these lines perfectly matched the expression pattern of endogenous Mmp1 as determined by *in situ* hybridization experiments (Uhlirova, M. & Bohmann, D., The EMBO Journal, 2006; Llano, E. *et al.*, J Biol Chem, 2000; Page-McCaw, A. *et al.*, Dev Cell, 2003). Therefore, we considered this element sufficient for the potential LPS effect studied in this project. To better illustrate our rationale, we extended the part of the manuscript that describes the selection of the promoter region for reporter construction. See lines 273-281 in the revised manuscript.

*Is it clear that the cells divide normally and are healthy (i.e. not apoptotic) after the LPS stimulus?*

**Response:** Exposure of S2 cells to LPS has been widely used as a cell culture model to study immune responses to microbial challenges. LPS pre-treatment has been restricted to a short exposure period of only 2 h in our multiple treatment experiments and LPS was subsequently washed out. In all our experiments, we included time-matched controls exposed to PBS only. We did not observe any difference between cells exposed to LPS or PBS regarding cell division rates or cell numbers after the pre-treatment and before re-exposure.

**In response to Reviewer 2:**

*Reviewer #2: Geigges et al set out to study the possible memorization of the innate immune response of Drosophila S2 cells to stimulation by bacterial LPS. Suspecting that the PRC epigenetic regulators may play a role in such a memory, they mined pre-existing genomic data for genes induced by LPS and at the same time are positioned near chromatin binding peaks of Pc, Ph and Psc. They studied four such Pc-target/LPS-induced genes, Mmp1, upd2, PGRP-LF, puc, and a non-Pc-target LPS-induced gene CecA by RT-qPCR after LPS stimulation. Disappointingly, the kinetics and amplitude of the transcriptional response of all these genes was not affected by prior exposure to LPS, whether single or multiple. Besides testing the original S2 cell line ("wt S2 cells"), they generated a subclone stably transformed with an Mmp1 promoter-GFP reporter construct to be able to study the population profile of the response to LPS by flow cytometry. Again, this line showed no difference in kinetics, amplitude or population profile of GFP expression whether it had been exposed to LPS before or not. The five genes studied in wt S2 cells were also studied in the reporter subclone and again no effect of prior LPS exposure was seen. Even when challenged with low doses of LPS, that produce a lower level of target gene induction, no differences were observed.*

*Since the target genes tested showed no sign of transcriptional memory in either of the cell lines, the role of the PRC could not be addressed. So, this paper gives us no insight on whether epigenetic regulators play a role in innate immune memory, since the authors were never able to observe such memory in the first place. The only conclusion that can be reached is that four Pc target immune response genes show no transcriptional memory to earlier immune stimulation, same as the non-Pc-target CecA. Even this conclusion needs one more experiment in order to be solid: The authors should confirm that these four genes are indeed in a Pc-chromatin domain in their new reporter cell line (and that CecA is not).*

**Response:** We agree with reviewer 2 that it is necessary to verify that the LPS-inducible PcG target genes are also targeted by the PcG system in the Mmp1 reporter cell line. Using chromatin immunoprecipitation (ChIP) followed by RT-qPCR, we were able to confirm that the Pc protein is bound to the loci of candidate genes including Mmp1 to a similar extent in the Mmp1 reporter cell line as in WT S2 cells. Thereby, we can rule out an altered non-PcG chromatin state in the reporter cell line as a reason for not finding a transcriptional memory of previous LPS exposures and can safely conclude that there is no PcG system-mediated memory of LPS stimulation for the target gens analyzed in this study. We describe this ChIP experiment in the results section of our manuscript (lines 347-351 in the revised manuscript) and more extensively in the Supporting Information. We have added a new figure (Fig S3) to present the ChIP results.

*An interesting observation of this paper is that wt S2 cells responded stochastically to LPS stimulation, only 25% of the cells responded. In contrast, in the reporter cell line 80-90% of the cells responded to LPS. Also the amplitude of response was higher in the reporter cell line (compared to wt S2 cells) for 3 out of the 4 Pc-target-genes. Perhaps this subclone has altered chromatin state at these genetic loci (they may have lost the Pc marks) and this may partially explain why no memory was observed. Granted, no memory was detected even in wt S2 cells, but, given the heterogeneity of their response to LPS, we cannot safely conclude that no memory existed on a cell-by-cell basis.*

**Response:** This is true. Due to the heterogeneous response to LPS in WT S2 cells, a solid conclusion about the potential memory of LPS exposure in WT S2 cells is not possible – as stated in our manuscript, lines 240-246. To circumvent this issue, we have generated the reporter cell line. As nearly all reporter cells responded to LPS, this cell line can be used to accurately analyze the cellular response to repeated LPS exposure. As shown with additional ChIP experiments (as described above), we can exclude an altered chromatin state at the analyzed LPS-inducible PcG target genes in the reporter cell line. Therefore, we can now safely conclude – as acknowledge by reviewer 2 – that a previous induction of the LPS-inducible PcG target genes analyzed in this study is not memorized in terms of an altered cellular response to a second stimulus.

*Another point that the authors could highlight is that the Pc targets show low levels of transcriptional induction, 2-20x, whereas CecA shows close to 1000x induction upon LPS treatment. Could their association with Pc chromatin dampen their level of induction?*

**Response:** We agree with reviewer 2 that this is an interesting point that we have not highlighted yet. Consequently, we have added this interesting thought to the discussion section, see lines 524-527 in the revised manuscript.

*In conclusion, whereas the experiments are well performed, controlled and clearly presented, they fall short of providing any insight on either Pc function or innate immunity memory. This negative result could be worth publishing if the chromatin state of the genes under study is determined in the cell line used, as described above.*

**Response:** We are happy to see that reviewer 2 shares our opinion that the experiments are well performed, controlled and clearly presented. We have addressed the point about the PcG chromatin state of the LPS target genes in the reporter cell line as discussed above in order to make our results worth publishing as requested by reviewer 2.
