# Supplementary material for: Priming exposures to lipopolysaccharides do not affect the induction of Polycomb target genes upon re-exposure
Source: PLoS One. 2020 Apr 14;15(4):e0231498. doi: 10.1371/journal.pone.0231498 (PMC7156044; doi:10.1371/journal.pone.0231498)
Supplement: Attachment — Submitted filename: revision_II_response_to_reviewers_final.docx [file pone.0231498.s003.docx]

**Revision of Manuscript PONE-D-19-32589R1 – Response to Reviewers**

We would like to gratefully thank both reviewers and the editor for their interest in our manuscript and their valuable contributions towards its improvement.

The manuscript underwent another revision to comply with further comments by one reviewer. Below, is our point-by-point response to reviewers’ comments.

**In response to Reviewer 1:**

*Reviewer #1: The manuscript has significantly improved in depth of analysis and clarity of presentation in the revision process. I am overall satisfied in the way the authors have handled my criticism and recommend publication.*

**Response:** We are happy to see that we could fulfill the requests of reviewer 1 in our thorough revision of the manuscript.

**In response to Reviewer 2:**

*Reviewer #2: The authors performed the ChIP experiments needed to confirm the Pc-bound state of two of the genes used in their study. However, the ChIP data presentation is not informative. The authors state (in the supplementary methods) that "results for each target locus were displayed as fold enrichment over negative control." I take this to mean IP(Mmp1)/ IP (non-target region near bx). The correct way to show the results would be to present IP(Mmp1)/input(Mmp1) and as a separate data point to present IP(near bx)/ Input (near bx). This would show the level of enrichment and would avoid the enormous apparent increase in Pc enrichment at the transgene (50x compared to the same region in wt S2 cells), which I strongly believe is simply due to higher copy number of the transgene with respect to the non-target locus in the denominator.*

**Response:** We fully agree with reviewer 2 that the ChIP data should be presented in a more informative manner. We have adapted Fig. S3 accordingly and now present the data as IP / input. As suggested, we added the data for the non-target locus as a separate data point in the modified figure. This representation of the data confirms the comment of reviewer 2 that the enormous increase in Pc enrichment at the transgene in the initial data representation is likely due to a higher copy number of the Mmp1 transgene with respect to the non-target locus in the denominator. In the modified Fig. S3, it becomes clear that the Pc protein is bound to the loci of candidate genes including Mmp1 to a similar extent in the Mmp1 reporter cell line as in WT S2 cells. In line with the new data representation, we have modified the information on ChIP data representation in the Supplementary Materials and Methods section of the Supporting Information, lines 244-245. Additionally, the legend of Fig. S3 was adapted to the new way of representing the data. See lines 53-59 of the Supporting Information.

*A couple more points that I noticed on rereading the manuscript:
(1) Suppl. Fig. 4: Panels D and E disagree. Based on the histograms in panel D, the mean GFP intensity (panel E) at the two earliest timepoints should show significant differences between pretreated and untreated cells.*

**Response:** It is correct that the mean GFP intensity of the whole cell population at the 0 min and the 30 min time points is different between non-pre-treated and pre-treated cells as we have stated in lines 96-100 of the Supporting Information. This information is clearly shown in Fig. S4D as acknowledged by reviewer 2. In Fig. S4E, however, we display the mean GFP intensity of the GFP-positive cell subset only (not of all cells) to make the point that the GFP intensity of the cells that are GFP-positive is the same in non-pre-treated and pre-treated cells, although the number of GFP-positive cells is different at the early time points. To emphasize this, we have added “GFP-positive cell subset” to the headline above Fig. S4E (see line 71). In addition to the information already present in the figure legend, this should make clear that Fig. S4E focuses on the subset of GFP-positive cells only.

*(2) Lines 326-330 describe an increased amplitude of response for Mmp1 in the GFP line compared to wt S2 cells. The most logical explanation is that this is simply a population effect, since 90% of the cells respond to LPS in the GFP line, compared to only 25% in the wt S2 (and the RpL32 gene used for normalization is expressed in all cells). This population effect is acknowledged in lines 520-523 in the Discussion. I would add a sentence or two (in the Discussion) to speculate on the other targets that do NOT show similar 4x higher induction levels in the reporter line vs wt S2 cells. Could it be that the 25% - 90% change in response between wt S2 cells and reporter line is particular to the Mmp1 gene and puc, PGRP-LF etc would show a more equivalent responding population?*

**Response:** We agree that this is an interesting argument that we have not considered yet, and thank the reviewer for pointing out this issue. Consequently, we have added this thought to the discussion section, see lines 523-527 in the revised manuscript.
